# A personalized molecular approach in multiple myeloma: the possible use of RAF/RAS/MEK/ERK and BCL-2 inhibitors

**DOI:** 10.37349/etat.2022.00095

**Published:** 2022-08-31

**Authors:** Vincenzo Raimondi, Nicolas Thomas Iannozzi, Jessica Burroughs-Garcìa, Denise Toscani, Paola Storti, Nicola Giuliani

**Affiliations:** 1 Department of Medicine and Surgery, University of Parma, 43126 Parma, Italy; 2 Hematology, “Azienda Ospedaliero-Universitaria di Parma”, 43126 Parma, Italy; Fondazione IRCCS Istituto Nazionale dei Tumori, Italy

**Keywords:** Multiple myeloma, mitogen-activated protein kinases pathway, B cell lymphoma 2, venetoclax

## Abstract

Multiple myeloma (MM) is a blood cancer that derives from plasma cells (PCs), which will accumulate in the bone marrow (BM). Over time, several drugs have been developed to treat this disease that is still uncurable. The therapies used to treat the disease target immune activity, inhibit proteasome activity, and involve the use of monoclonal antibodies. However, MM is a highly heterogeneous disease, in fact, there are several mutations in signaling pathways that are particularly important for MM cell biology and that are possible therapeutic targets. Indeed, some studies suggest that MM is driven by mutations within the rat sarcoma virus (*RAS*) signaling cascade, which regulates cell survival and proliferation. The *RAS*/proto-oncogene, serine/threonine kinase (*RAF*)/mitogen-activated extracellular signal-regulated kinase (*ERK*) kinase (*MEK*)/*ERK* signaling pathway is deregulated in several cancers, for which drugs have been developed to inhibit these pathways. In addition to the signaling pathways, the disease implements mechanisms to ensure the survival and consequently a high replicative capacity. This strategy consists in the deregulation of apoptosis. In particular, some cases of MM show overexpression of anti-apoptotic proteins belonging to the B cell lymphoma 2 (*BCL-2*) family that represent a possible druggable target. Venetoclax is an anti-*BCL-2* molecule used in hematological malignancies that may be used in selected MM patients based on their molecular profile. We focused on the possible effects in MM of off-label drugs that are currently used for other cancers with the same molecular characteristics. Their use, combined with the current treatments, could be a good strategy against MM.

## Introduction

**Figure 1.**
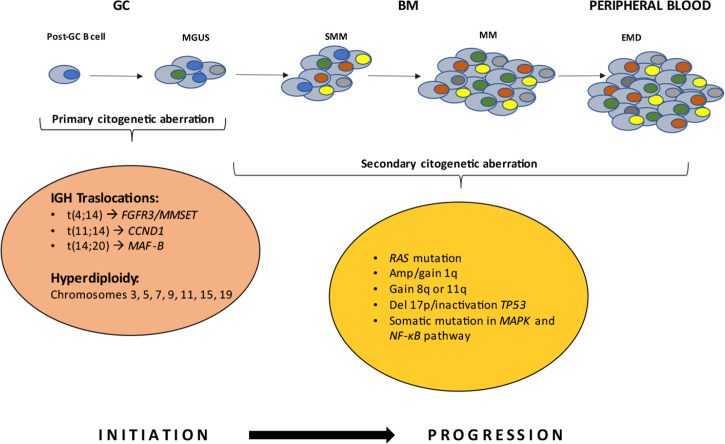
The basis of neoplastic transformation and development of MM are mutational events at the PC level. Early genetic events drive the MGUS stage. Further mutations induce deregulations in the PCs, leading to the transition to the SMM and MM stages. The latter steps are characterized by the proliferation of monoclonal PCs in the BM microenvironment. In rare cases, it can evolve into extramedullary disease (EMD). *FGFR3*: fibroblast growth factor receptor 3; *MMSET*: multiple myeloma SET domain; *CCND1*: cyclin D1; *MAF-B*: musculoaponeurotic fibrosarcoma oncogene homolog B; Amp: amplification; Del 17p: deletion 17p; *TP53*: tumor protein p53

Multiple myeloma (MM) is a hematological malignancy, with an annual incidence of approximately 6.6 cases/100,000 people [1]. It is a plasma cells (PCs) neoplasm, which provides the function of secreting antibodies in the bone marrow (BM). This tumor is usually diagnosed by serum protein electrophoresis (SPEP), in which a distinctive M peak is evident, or by estimation of free light chains in urine [2]. MM cells are the malignant counterparts of long-lived PCs in the post-germinal center (GC), characterized by strong BM dependence and somatic hypermutation (SHM) of immunoglobulin (Ig) genes [3]. MM is affected by in-depth genetic alterations that occur during the progression of the disease, which is why it is generally recognized as a highly heterogeneous disease. Its evolutionary course starts with a pre-malignant status noted as monoclonal gammopathy of undetermined significance (MGUS) [4], a posterior stage termed smoldering MM (SMM) [5], and may ultimately escalate into symptomatic myeloma [2]. The hallmark genetic events (or aberrations) are commonly classified as primary and secondary events. Primary aberrations are grouped into two subgroups: non-hyperdiploidy, comprised of primary Ig (t) translocations involving the Ig heavy chain (IGH) in the 14q32 region including t(4;14), t(11;14), t(14;20), and the hyperdiploidy group comprised of trisomies of chromosomes 3, 5, 7, 9, 11, 15, 19 [6]. Multi-copy chromosomes carry genes whose overexpression may be responsible for the replicative potential of cells. The MGUS clone is subsequently affected by secondary abnormalities that may or may not lead to the transition to the MM [7]. Secondary abnormalities, also genetic, include MYC proto-oncogene (*MYC*) translocation, 17p or 1p32 chromosome deletion, 1q chromosome amplification or mutations of rat sarcoma virus (*RAS*) gene [NRAS proto-oncogene (*NRAS*) and KRAS proto-oncogene (*KRAS*)], B-Raf proto-oncogene (*BRAF*), mitogen-activated protein kinase (*MAPK*), and nuclear factor kappa-light chain enhancer of activated B cells (*NF-κB*) pathways or overexpression of the anti-apoptotic protein B cell lymphoma 2 (*BCL-2*) [8] (Figure 1).

### Primary genetic aberrations

The most common translocation in MM, impacting 15% of patients, is t(4;14)(p16;q32), and is associated with a very unfavorable prognosis [9]. Translocation t(4;14) results in the overexpression of two key genes, *FGFR3* and *MMSET*, also known as nuclear receptor-binding SET domain 2 (*NSD2*) or Wolf-Hirschhorn syndrome candidate 1 (*WHSC1*). Overexpression of *MMSET* isoforms is a universal feature of t(4; 14) [10–12]; whereas the same is not true for *FGFR3*, as approximately 30% of patients affected by MM with t(4;14) do not express *FGFR3*. Transforming activity of this gene has been reported both *in vitro* and *in vivo*.

The t(11;14)(q13;q32), is detected in 15% of MM patients. Translocation can be observed in the condition of MGUS. This genetic event results in the over-regulation of nuclear *CCND1* [13]. An intriguing aspect is that patients diagnosed with this translocation can remain stable in the MGUS status with no disease progression for decades. On this basis, the outcome can be assumed to be therefore favorable.

Translocation t(14;20) is diagnosed in 3% of patients with MM [14]. It is associated with a poor prognosis and, as a result of chromosome aberration, there is an overexpression of the *MAF-B* gene [15, 16].

### Secondary genetic aberrations

The disease evolves along with secondary genetic disorders, including *MYC* translocation, which can occur in ~15% of MM at diagnosis and 50% of more severe stages [17]. The *MYC* gene codifies an extremely important transcription factor engaged in various cellular functions such as cell growth [18], proliferation [19], and protein synthesis [20]. *MYC* is implicated in the enumerated biological processes and plays a cardinal role in tumor advancement. In MM patients, its overexpression is characteristically linked to a poor prognosis.

As a secondary genetic event, it is also good to focus on copy number variations (CNVs), which are a hallmark of MM progression. This genetic event consists of DNA gain or loss at a whole chromosome arm, a whole chromosome, or a focal region [21]. It is a disorder typically known as aneuploidy and discriminates cancer cells from their benign counterparts. CNVs are detected in approximately 70% of MM cases [22].

CNV-containing PCs have a selective advantage in promoting disease progression because the amplified or deleted regions contain significant genes involved in the development and progression of MM [23]. The gold standard technique to detect selected CNVs is fluorescence *in situ* hybridization (FISH), but its results are limited to the probes used in each analysis. Some centers also use the single nucleotide polymorphism (SNP) microarray to reveal copy number alterations at a higher resolution.

Yellapantula et al. [24] used a custom next-generation sequencing (NGS) acquisition panel specifically designed to help the identification of rearrangements in MM. In their study, they reported high sensitivity (> 99%) and specificity (> 99%) for revealing chromosomal gains and losses [24].

The CNVs most commonly found in MM are on chromosomes 1, 13, 17. On chromosome 17, it is important to evaluate the role of *TP53* gene, which has cancer suppressor function. In particular, chromosome 17p can be deleted in MM. This peculiar genetic aberration is also claimed to be a secondary event in disease progression [25]. Biallelic *TP53* inactivation is accepted as an advanced driver in MM and has been most closely associated with low overall survival and resistance to standard treatments. It is found in 3.7% of patients [26]. In MM, *TP53* mutations pose late events in disease progression; in fact, this mutation is rarely observed at diagnosis, demonstrating a central role in the progression of MM [27].

Another chromosome susceptible to CNVs is chromosome 13, which undergoes a deletion at arm q. The deletion is found in 50% of newly diagnosed MM patients.

Initially, this genetic event was associated with a poor prognosis. Over time, however, this view has changed, as recent studies have shown that the deletion coincides with other remarkable genetic events such as t(4;14). Thus, establishing the correct prognosis associated with this deletion remains a challenge. There are about 68 genes influenced by the deletion of the region 13q, including RB transcriptional corepressor 1 (*RB1*), EBP like (*EBPL*), ribonuclease H2 subunit B (*RNASEH2B*), RCC1 and BTB domain containing protein 2 (*RCBTB2*), and the microRNAs microRNA-16-1 (*miR-16-1*) and *miR-15a*. The region containing these genes is 13q14.11-13q14.3 [28]. Among these genes, one with a special role in cell cycle regulation is *RB1*. The miRNA cluster containing *miR-16-1* and *miR-15a*, instead, seems to be deleted in chronic lymphocytic leukemia and regulates the expression of several cell cycle genes [29].

An incisive role in the progression of MM is assumed by chromosome 1q, particularly the region 1q21. In 40% of *de novo* cases and 70% relapsed-refractory MM (RRMM) patients, gain and/or amplification of chromosome arm 1q21 (1q21+) is detected [30, 31]. The presence of 3 copies of this region is defined as gain, while ≥ 4 copies characterize amplification. Chromosome amplification 1q (1q21 amp) is one of the most common secondary cytogenetic abnormalities in patients with MM. Moreover, not all the genes contained in this region have been identified, but those that are thought to be determinants in the disease development include *BCL-9*, cyclin-dependent kinase regulatory subunit 1B (*CKS1B)*, interleukin-6 receptor (*IL6R*), interleukin-2 enhancer binding factor 2 *(ILF2*), and myeloid cell leukemia 1 (*MCL-1*). 1q21 amp is often associated with drug resistance, disease progression, and poor prognosis [32].

### Deregulated molecular pathways in MM

The pathogenesis of MM also highlights changes occurring in the BM microenvironment [33]. Genetic defects identified in the tumor and interactions between MM and BM microenvironment cells lead to the activation of signaling pathways that enhance the expansion of malignant clones and stimulate two phenomena essential for MM progression, namely neo-angiogenesis and osteoclastogenesis [34, 35]. Over-regulated signaling pathways are represented by the *RAS/RAF/MEK/ERK*-(also known as *MAPK*-) pathway, the phosphatidylinositol 3-kinase (*PI3K*)*/*protein kinase B (*AKT*)- and *NF-κB*-pathways, but also the Janus kinase (*JAK*)*/*signal transducers and activators of transcription (*STAT*)*-, Hedgehog-, Notch-,* transforming growth factor beta (*TGFβ*)*-,* and Wingless-related integration site (*WNT*)- pathways. The *RAS/RAF/MEK/ERK* pathway consists of a cascade of intracellular proteins with kinase activity involved in cell proliferation, growth, adhesion, and apoptosis [36]. *RAS* [*NRAS, KRAS,* and HRAS proto-oncogene (*HRAS*)] activates *RAF*-kinases, which, in turn, phosphorylate *MEK* and finally *ERK*-kinase. Many cancers share mutations in protein kinases involved in this pathway. Therefore, one strategy implemented in oncology has been to target regulators of this pathway, as in colorectal cancer, hairy cell leukemia, and melanoma. The percentage of MM patients presenting with mutations in the *MAPK* pathway are 43–53%, thus resulting in a fairly common gene event in this disease [37–39].

Thus, in this review we will focus on the *RAS/RAF/MEK/ERK* signaling pathway and the role of the anti-apoptotic protein *BCL-2*, both of whom are engaged in the pathogenesis of MM and discuss the implementation of related targeting therapies. In fact, to survive the numerous genetic insults and pro-apoptotic changes, malignant cells must upregulate anti-apoptotic *BCL-2* proteins and thus become highly dependent on the activity of these proteins [40]. Therefore, *BCL-2* may also represent a therapeutic target in the struggle against MM.

## Off-label prescriptions

Off-label drug use refers to the use of drugs under conditions other than those for which they were approved, such as dose, age of the patient, administration route, and contraindications [41]. This strategy is implemented to treat health problems for which there are no other currently approved drugs, for example in the case of uncommon diseases or specific subset of patients [42]. Off-label uses may prove particularly valuable in treating patients with an orphan disease, for which it may be the only available treatment [43].

The purpose of off-label use is to aid in the recovery of an individual patient. In oncology, pediatrics, geriatrics, and obstetrics, patient care can be difficult if off-label use is not employed [44, 45]. An estimate claims that off-label prescribing reaches 90% in the pediatric population or 40% in adults [46]. In oncology it achieves up to 50% of patients, and is widely applied in pediatric oncology [47]. The use of off-label drugs is believed to have contributed to an overall cure rate of more than 70% in pediatric malignancies [48]. Underlining, consequently, that cancer patients can benefit greatly from this phenomenon. One issue raised, however, is that the use of off-label drugs is not always supported by solid scientific evidence [41]. Scientific review and investigation of medications, that are tested and approved for a given condition, help protect the patient as much as possible from side effects. By using off-label drugs, this protection may be compromised [49], so they should be used in a precise and controlled manner.

Several patients with different tumors expressing the same target therefore may benefit from off-label prescription [50]. This concept is carefully translated into MM. Accordingly, it is possible to employ a variety of off-label drugs that can block the de-regulated molecular pathways in this cancer.

## Targeting the MAPK pathway in MM

The *RAS/RAF/MEK/ERK* pathway is a critical intermediary of many essential biological processes. It is involved in cell proliferation, migration, survival, and angiogenesis. Members of the RAS protein subfamily, NRAS, KRAS, and HRAS, function as molecular switches in cellular signal transduction. These small guanosine triphosphate hydrolases (GTPases) activate RAF-kinases, which in turn phosphorylate *MEK*s and finally ERK-kinases.

*MAPK*-pathway mutations are one of the most common mutations found in MM [37]. It is also intriguing to note that the number of patients with mutations appears to be higher in relapsed disease [39]. The literature data on the prognostic significance of mutations on the *MAPK* pathway do not follow a univocal trend but, on the contrary, seem to be conflicting. While some research groups have found negative effects of *NRAS* mutations [51], others have observed a negative effect in *KRAS* mutations [52], and still others have found no prognostic effect [53]. Despite the high prevalence of mutations activating the *MAPK* pathway, these controversial results can be explained by considering that only some of the mutations effectively appear to activate it.

**Figure 2.**
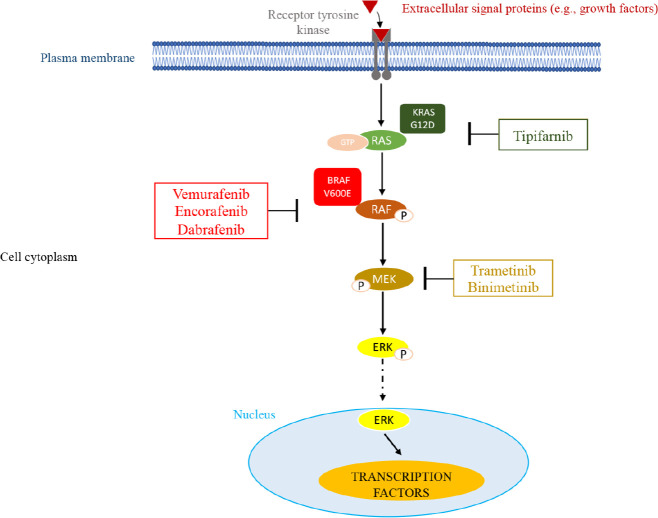
*MAPK* pathway and potential sites of therapeutic intervention with inhibitors. This molecular pathway is triggered by various extracellular signals. The main components of this pathway are RAS, RAF, and MEK leading to the activation of ERK through its phosphorylation (P). Once activated, ERK migrates into the nucleus where it activates transcription factors that affect cell proliferation and survival. In myeloma, overactive *RAS/RAF/MEK/ERK* signaling resulting from genetic mutations in the RAS and BRAF GTPases can be targeted by small molecule inhibitors of RAS G12D (tipifarnib), BRAF V600E (vemurafenib, encorafenib, dabrafenib), or *MEK* (trametinib, binimetinib)

It has been previously demonstrated that only KRAS G12D and BRAF V600E consistently resulted in phosphorylation of the ERK downstream target (Figure 2). Other mutations were associated with increased phospho-ERK levels in only a small percentage of cases.

As a consequence of the difficulty in targeting RAS, to date only one specific inhibitor has been identified for the KRAS G12C mutation, but this is rare in MM [54]. An investigation was conducted using the inhibitor tipifarnib, which was found to inhibit RAS and to have limited activity in MM patients. It also does not diminish, significantly, the activation of the *MAPK* pathway [55]. Along the lines of what was observed, the emphasis shifted to *MAPK* pathway inhibitors having downstream targets of RAS, which include BRAF and MEK.

Due to several pitfalls, it is necessary to accomplish patients screening. In a number of malignancies, it has been noted how, in the absence of the mutation involving *BRAF*, treatment of patients with inhibitors of this mutation can drive an increase in RAS signaling those results in activation of the pathway. This effect would appear to be triggered by a decrease in negative feedback at the level of RAS [56], binding of wild-type BRAF to CRAF, and subsequent *MAPK* signaling through *CRAF* [57, 58].

Attention must also be paid to how to identify patients who benefit from inhibition of the *MAPK* pathway [59]. A retrospective study that examined the effects of treatment with trametinib (MEK 1/2 inhibitor) illustrates this problem well. In that study, patients with mutations determining *MAPK* pathway triggering or carrying oncogenic mutations in *NRAS*, *KRAS*, and *BRAF* were selected. Of this cohort of patients, only 40% achieved at least a partial response (PR) when treated with trametinib coupled with other drugs, whereas only 10% exhibited at least a PR when treated solely with trametinib [60].

This underscores the critical importance, in terms of therapeutic efficacy, of precisely identifying patients who might be eligible for *MAPK* pathway inhibition. Several recent studies have therefore included only patients with the BRAF V600E/K mutation, which has been shown to consistently activate ERK and has also been closely examined in a variety of other cancers. In addition, several potent BRAF V600E mutation-specific inhibitors are available, such as vemurafenib, encorafenib, and dabrafenib. Approximately 5% of patients harbor a BRAF V600E clone or subclone. Early data on targeting the BRAF V600E mutation in patients with MM have been contradictory: some studies have shown treatment efficacy in RRMM patients with the mutation [61, 62], while others did not detect any response [63, 64].

Although BRAF targeting is effective in tumor types carrying mutant BRAF, however, there is rapid resistance against BRAF inhibitors. This phenomenon can be explained by several mechanisms among which one of the most frequent involves the acquisition of activating mutations upstream or downstream of *BRAF* in *NRAS* or *MEK*, leading to alternative signaling and bypass of *BRAF* [65, 66]. In addition, MEK inhibition has been seen to induce therapeutic resistance through upregulation of other signaling pathways, such as the *PI3K/AKT* pathway [67]. To circumvent these resistance mechanisms, it has been suggested to combine BRAF inhibition with MEK inhibition in a way that acts on two of the interactors involved in the signaling pathway. These approaches have been found to be very efficient in melanoma cases and, notably, dual inhibition has been more effective than BRAF inhibition alone [68]. Currently, dual inhibition is the most widely used strategy in MM studies.

### Clinical trials

The BIRMA trial examined the inhibition of BRAF and MEK in MM patients carrying the BRAF V600E/K mutation using a combination of encorafenib and binimetinib (a selective inhibitor of MEK). Preliminary results from this study showed an overall response rate (ORR) of 82% with 9 of 11 patients having at least one PR. This study demonstrated that, at least for some patients with MM, pathway inhibition leads to clinically meaningful responses [69].

Regarding the BRAF/MEK co-inhibitor, according to a study by Haertle et al. [70], proteosome inhibitors were ineffective with *RAF/NRAS/KRAS* activating mutations. In contrast, co-treatment of bortezomib and BRAF/MEK co-inhibitor had a synergistic action in the presence of activating mutations.

Another trial (NCT03091257), tests dabrafenib and/or trametinib in patients with RRMM. The purpose of the study is to examine the efficacy of BRAF inhibition in mutation-positive patients and the effects of MEK inhibition in patients with only *RAS* mutations.

Thus, we can state that inhibition of the *MAPK* pathway shows promising activity in a subset of patients. Therefore, a preliminary assessment of the presence of mutations involved in this pathway could guide decision-making on therapeutic strategies.

## BCL-2 family proteins and their targeting in MM

The *BCL-2* gene family encodes more than 20 proteins that regulate the intrinsic apoptosis pathway and are fundamental to the balance between cell survival and death [71].

The BCL-2 family proteins can be categorized into three groups, namely antiapoptotic multidomain proteins (such as BCL-2, BCL-XL, and MCL-1), proapoptotic multidomain proteins [like BCL-2-associated X (BAX), BCL-2 antagonist/killer (BAK), and BCL-2-related ovarian killer (BOK)], and proapoptotic BCL-2 homology domain 3 (BH3)-only members [e.g., P53 up-regulated modulator of apoptosis (PUMA), NOXA, BH3 interacting-domain death agonist (BID), and BLC-2 interacting mediator of cell death (BIM)] [71].

BCL-2 family proteins are capable of giving rise to different interactions; however, their corresponding interaction turns out to be selective and specific. For example, BIM and PUMA can bind all members of the anti-apoptotic multidomain BCL-2 family, whereas NOXA binds only to MCL-1 [72, 73]. In contrast, BCL-2 associated agonist of cell death (BAD) interacts with BCL-XL and BCL-2, but not with MCL-1 [72, 74].

The intrinsic pathway involves the mitochondria and after receiving the stimulus, the pro-apoptotic BH3-only members bind and neutralize the antiapoptotic proteins. This leads to oligomerization of multi-domain pro-apoptotic member (BAX/BAK) present on mitochondrial membrane surface whose activation leads to permeabilization and formation of pores in outer mitochondrial membrane, releasing various apoptotic mediators [high-temperature requirement protein A2 (HtrA2), also called Omi, second mitochondria-derived activator of caspase (Smac)/direct inhibitor of apoptosis (IAP)-binding protein with low pI (DIABLO), cytochrome c, endonuclease G (Endo G) and apoptosis-inducing factor (AIF)] [75]. BAK may contribute to early mitochondrial fragmentation while BAX is probably more important for subsequent pores development and degeneration in the outer membrane [75]. The release of cytochrome c in cytosol causes the association of apoptosis protease-activating factor 1 (APAF-1) and ATP/dATP to form intracellular apoptosome that activates caspase-9 [76]. Disrupted mitochondria also produce Smac/DIABLO, which releases caspase-3 from X-linked IAP (XIAP)-mediated inhibition. The role of IAP is to act a guardian inside a cell to defend against the mediator of apoptosis (HtrA2/Omi, Smac/DIABLO) by binding to caspase-3/-9 whereas Endo G and AIF operate independently of caspase causing chromatin condensation and fragmentation. Anti-apoptotic members of the BCL-2 family, through direct protein interactions involving binding to their BH3 motifs, inhibit the activity of BAK/BAX. Inhibition of the anti-apoptotic members of this protein family has been accepted in clinical practice being that BCL-2 family members are key regulators of common apoptotic pathways [77].

Several BH3 mimetics have entered clinical trials [78], although, due to the absence of a trustworthy validation assay to directly test the mitochondrial activity of new candidate BH3 mimetics, there have been many erroneous reports of agents advertised as BH3 mimetics despite their off-target mechanisms of action. BH3 profiling assesses the activity of a compound at the mitochondrial level by measuring cytochrome c release as a marker for mitochondrial outer membrane permeabilization.

Villalobos-Ortiz et al. [79] proposed a comprehensive biochemical toolkit consisting of BH3 profiling in conjunction with the high-throughput Annexin V/Hoechst viability assay for validation of BH3 mimetic candidates. In order to accredit mitochondrial assays of BH3 mimetics, they performed a BH3 profiling method (iBH3) that consists of exposing mitochondria to standardized concentrations of BH3 peptides and measuring outer mitochondrial membrane permeabilization by cytochrome c release using flow cytometry [79].

Another approach was proposed by Bhola et al. [80] who developed a high-throughput method to assess the sensitivity to BH3 mimetics of tumors within 24 h of excision, providing data demonstrating that the very process of prolonged *ex vivo* propagation rapidly alters chemical vulnerabilities relative to the primary tumor [80].

The same authors nicely demonstrated a relationship between BCL-2 pathway and RAS signaling in cancer stem cell providing the rational to design new strategy to treat resistant cancers [81].

**Figure 3.**
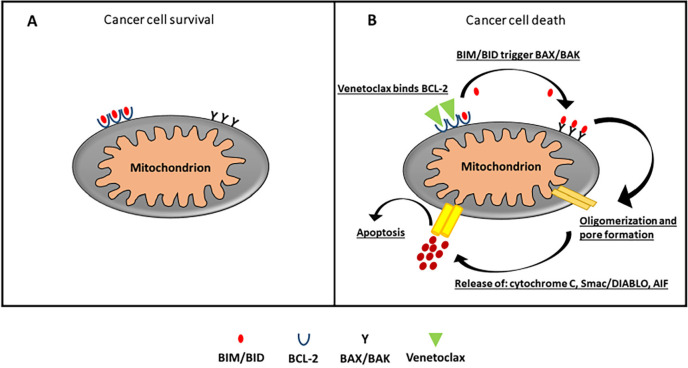
A. In cancer cells, excessive production of BCL-2 sequesters and blocks the function of pro-apoptotic protein and evades apoptosis. B. Venetoclax, a selective BCL-2 inhibitor, displaces and reactivates pro-apoptotic proteins bound to the BCL-2 binding groove. This prompts to oligomerization of BAX/BAK whose activation leads to permeabilization and formation of pores in outer mitochondrial membrane, releasing cytochrome c, Smac/DIABLO, and AIF

Drugs mimicking the action of BH3-only proteins indirectly lead to BAX/BAK activation. This allows permeabilization of the mitochondrial outer membrane, apoptosome formation and subsequent caspase activation and apoptosis. This is particularly true in the treatment of chronic lymphatic leukemia, typified by the approval of the BCL-2 inhibitor venetoclax as a particularly effective rescue therapy for this disease [70]. The ability of venetoclax is to bind BCL-2, through high-affinity binding, causing blockade of its signaling in the cell; this is the mechanism of action that is responsible for the *TP53*-independent apoptotic event [82] (Figure 3).

Chronic lymphocytic leukemia cells can effectively evade apoptosis due to their marked dependence on BCL-2 activity; in other malignancies, distinct members of the BCL-2 family by contrast may have a greater influence. This is the situation in MM. Indeed, in PC and MM, previous preclinical studies have indicated that MCL-1 may be the major anti-apoptotic analog of BCL-2 [8]. Based on this, venetoclax being specific for BCL-2, its clinical trial in MM patients has begun with moderate enthusiasm.

However, it has been demonstrated in MM cell lines and in primary patient samples that venetoclax is highly effective in a specific subgroup of MM with t(11;14), which is present in approximately 20% of MM, mainly because of the higher BCL-2/MCL-1 messenger RNA (mRNA) ratio [83].

Nevertheless, even among patients with t(11;14) myeloma, the response rate is only 40–60% [84]. Knockdown of *CCND1* has been shown to fail to induce resistance to venetoclax, suggesting that t(11;14) and *CCND1* have no direct roles in response to venetoclax [85].

To further explore the biology responsible for venetoclax sensitivity in myeloma and to potentially identify additional biomarkers, Gupta and colleagues [85] performed a data analysis based on assessment of RNA-sequencing and assay for transposase-accessible chromatin (ATAC)-sequencing from samples of patients screened for venetoclax sensitivity. The identical analysis was also conducted using cell lines. Venetoclax-sensitive myeloma is enriched in B-cell-associated genes that are not typically expressed in PCs [85, 86]. The expression of these genes could not be explained completely by t(11;14) because such genes were observed almost exclusively in venetoclax-sensitive t(11;14) and were mostly absent in venetoclax-resistant t(11;14). No single gene was consistently expressed in all sensitive cell lines or patient samples, suggesting that a panel of genes or cell surface markers may be required to distinguish sensitive and resistant myeloma.

Through ATAC-sequencing analysis, they also identified a pattern of B-cell-like chromatin accessibility in venetoclax-sensitive cell lines, suggesting increased binding of transcription factors at some of these accessible sites. Overexpression of one of these transcription factors, basic leucine zipper ATF-like transcription factor (*BATF*), led to increased sensitivity to venetoclax. *BATF* is an essential B-cell developmental transcription factor which is activated during the B-cell receptor signaling, with subsequent activation-induced deaminase expression and class change recombination. However, its expression and role in PCs are not understood [87]. *BATF* represses and promotes transcription depending on the presence of other transcription factors, including the PC transcription factor interferon regulatory factor 4 (*IRF4*), and may contribute to the venetoclax-sensitive transcription program in PCs through alterations in chromatin accessibility. Thus, despite possessing the characteristics of terminally differentiated PCs, venetoclax-sensitive myeloma aberrantly preserves or reactivates aspects of the B-cell program, including dependence on BCL-2 that is normally downregulated during differentiation.

Although both *BCL-2* expression and the BCL-2/BCL-XL ratio are higher in venetoclax-sensitive cell lines and patients, they show a significant degree of overlap when comparing sensitive and resistant samples, thus making it difficult to select a specific cut-off that is useful for clinical decision-making [84, 88].

### Clinical trials

In the phase I clinical trial NCT01794520, venetoclax monotherapy was administered to patients with RRMM. Enrolled patients represented a heavily pre-treated group with a median of five prior lines of therapy. During the trial, the preclinical hypothesis [83] that myeloma patients with t(11;14) translocation responded more favorably to this therapy was confirmed. Therefore, this specific cohort was increased to include 30 patients. Compared with the entire study population (66 patients), the ORR was 21%; however, almost all of the patients who responded were from subgroup t(11;14). In this subgroup, the response rate amounted to 40%, which included 14% complete remissions (CRs). In contrast, only 2 of those patients without t(11;14) in the entire population of 66 individuals had a significant response to venetoclax [89].

When considering venetoclax as a targeted therapy for MM with t(11;14) and comparing it to other available treatment options for a population of heavily pre-treated RRMM patients, the use of venetoclax may emerge as an attractive salvage treatment option for these patients.

To expand the range of applicability of this approach the association between BCL-2 inhibition and other therapeutic modalities. Among its function, it is well established that bortezomib, a highly effective proteasome inhibitor for myeloma, can stabilize NOXA, an MCL-1 neutralizing protein [90].

Based on the above, being that the expression of MCL-1 may be a potential mechanism that triggers resistance to venetoclax activity, bortezomib has the potential to prove useful in combination with venetoclax. Apart from bortezomib, dexamethasone, a glucocorticoid used in therapeutic treatment in myeloma patients, has been shown to sensitize primary cells and myeloma lines to venetoclax activity. Based on this evidence, clinical trial M12-901 (NCT01794507) was initiated to test the triple combination venetoclax-bortezomib-dexamethasone in patients with RRMM [88]. Here, the response level reached 67% and was not limited to patients with t(11;14). The ORRs were similar in the different cytogenetic profiles; in particular, it was 47% in those patients who had the 17p deletion, a particularly high-risk subgroup with a severely impaired apoptosis process. It is important to note that higher levels of BCL-2 were observed in the subset of patients who had only 1–3 prior lines of therapy as compared to those with more than 4 lines of therapy [88].

These very promising early phase data prompted the initiation of clinical trial M14-031 (BELLINI), which aims to register the combination venetoclax-bortezomib-dexamethasone *versus* placebo-bortezomib- dexamethasone as a life-saving therapy for RRMM with 1–3 prior lines of therapy. Importantly, outcomes in patients with high-risk cytogenetics were distinct based on t(11;14) status and *BCL-2* gene expression. Trends in progression-free survival and overall survival favored the venetoclax arm in patients with either t(11;14) or *BCL-2* high gene expression regardless of cytogenetics status. In contrast, patients with high-risk cytogenetics and *BCL-2* low gene expression in the absence of t(11;14) were most at risk when treated with venetoclax due to the higher risk of toxicity. The most common Grade 3/4 adverse events (AEs) were neutropenia (21% *vs.* 8%), thrombocytopenia (15% *vs.* 30%), anemia (16% *vs.* 15%), diarrhea (15% *vs.* 12%), and pneumonia (18% *vs.* 13%). There were 12 treatment-emergent AEs (TEAEs) leading to death in the venetoclax arm and 1 in the placebo arm. Deaths attributed to infections were more common in patients treated with the combination of venetoclax and bortezomib [91].

Therefore, ongoing venetoclax-based studies are focusing primarily on patients with t(11;14) and/or *BCL-2* overexpression, marking the first biomarker-driven approach in MM. Based on these initial clinical data, it is conceivable that the inhibition of BCL-2 by venetoclax provided us with the entry point for individualized targeted therapy for a certain important subset of patients with MM. However, the combination bortezomib-venetoclax should be considered with caution in RRMM patients because of the high risk of infections and related toxic deaths.

The role of venetoclax will be further explored in the phase III CANOVA study (NCT03539744), which is evaluating the combination of venetoclax an dexamethasone *versus* pomalidomide and dexamethasone for t(11;14)-positive RRMM patients [92].

Importantly, as has already been established in another hematological malignancy (mutations in the *BCL-2* family) [93], the use of venetoclax can lead to the development of acquired mutations and, consequently, to treatment resistance.

So, in fact, as with other therapies, most patients with MM who initially respond to venetoclax eventually relapse. Many of these acquired resistances may occur through changes in cellular dependence from BCL-2 to MCL-1 or BCL-XL [94].

## Conclusion and perspective

MM is accompanied by high genomic instability reflected in multiple genetic abnormalities involving several cancer pathways; therefore, MM is typically characterized by high heterogeneity. The progress to discover sensitive genomic targets against which to develop novel agents with the potential to improve patient survival has made remarkable progress. However, these efforts have been severely challenged by the intricate biology of the disease and the molecular mechanisms of MM.

Despite all the progress, MM is still incurable in most patients. Future hopes are therefore directed toward identifying additional therapeutic strategies and new targets for its treatment.

In this review, we summarized the *RAS/RAF/MEK/ERK* and *BCL-2* signaling pathway describing their involvement in the pathogenesis of MM and the therapeutic approaches developed on them.

The efficacy of these therapeutic agents was tested on different MM cell lines and in MM patients and produced encouraging results. MEK inhibitors in combination with other kinase or mutant gene inhibitors have shown promising results in patients with RRMM. The combination delays the onset of acquired resistance resulting in increased progression-free survival and overall patient survival.

Similarly, the BCL-2 inhibitor venetoclax as monotherapy and in combination with other anti-myeloma agents has demonstrated improved outcomes in early clinical trials in MM patients with BCL-2 overexpression regarding the cytogenetic status.

With the availability of different types of treatment, over time it will be more complex to choose which treatment approach is most suitable for the patient and, as a result, it becomes necessary to understand how to incorporate such strategies into existing treatment approaches. An appropriate option might be to use a different combination of pathway-specific drugs that, by interacting with each other, could reduce the likelihood of developing drug resistance.

This could be further aided through deep molecular analysis of MM cells profile of the single patient, which would allow the identification of patients who would benefit from these drugs.

## Abbreviations

AIF: apoptosis-inducing factor
BAK: B cell lymphoma 2 antagonist/killer
BATF: basic leucine zipper ATF-like transcription factor
BAX: B cell lymphoma 2-associated X
BCL-2: B cell lymphoma 2
BH3: B cell lymphoma 2 homology domain 3
BM: bone marrow
BRAF: B-Raf proto-oncogene
CCND1: cyclin D1
CNVs: copy number variations
DIABLO: direct inhibitor of apoptosis protein (IAP)-binding protein with low pI
ERK: extracellular signal-regulated kinase
FGFR3: fibroblast growth factor receptor 3
IAP: inhibitor of apoptosis protein
Ig: immunoglobulin
KRAS: KRAS proto-oncogene
MAPK: mitogen-activated protein kinase
MEK: mitogen-activated extracellular signal-regulated kinase kinase
MGUS: monoclonal gammopathy of undetermined significance
MM: multiple myeloma
miR-16-1: microRNA-16-1
MYC: MYC proto-oncogene
NRAS: NRAS proto-oncogene
ORR: overall response rate
PCs: plasma cells
PR: partial response
RAF: RAF proto-oncogene, serine/threonine kinase
RAS: rat sarcoma virus
RRMM: relapsed-refractory multiple myeloma
Smac: second mitochondria-derived activator of caspase
TP53: tumor protein p53

## Declarations

### Copyright

© The Author(s) 2022.

## References

[1] Becker N. Epidemiology of multiple myeloma. Recent Results Cancer Res. 2011;183:25–35. 10.1007/978-3-540-85772-3_2 21509679

[2] Palumbo A Anderson K. Multiple myeloma. N Engl J Med. 2011;364:1046–60. 10.1056/NEJMra1011442 21410373

[3] Chesi M Bergsagel PL. Molecular pathogenesis of multiple myeloma: basic and clinical updates. Int J Hematol. 2013;97:313–23. 10.1007/s12185-013-1291-2 23456262 PMC3962846

[4] Kyle RA Therneau TM Rajkumar SV Offord JR Larson DR Plevak MF A long-term study of prognosis in monoclonal gammopathy of undetermined significance. N Engl J Med. 2002;346:564–9. 10.1056/NEJMoa01133202 11856795

[5] Manier S Salem KZ Park J Landau DA Getz G Ghobrial IM. Genomic complexity of multiple myeloma and its clinical implications. Nat Rev Clin Oncol. 2017;14:100–13. 10.1038/nrclinonc.2016.122 27531699

[6] Zhan F Huang Y Colla S Stewart JP Hanamura I Gupta S The molecular classification of multiple myeloma. Blood. 2006;108:2020–8. 10.1182/blood-2005-11-013458 16728703 PMC1895543

[7] Landgren O. Advances in MGUS diagnosis, risk stratification, and management: introducing myeloma-defining genomic events. Hematology Am Soc Hematol Educ Program. 2021;2021:662–72. 10.1182/hematology.2021000303 34889381 PMC8791104

[8] Gomez-Bougie P Amiot M. Apoptotic machinery diversity in multiple myeloma molecular subtypes. Front Immunol. 2013;4:467. 10.3389/fimmu.2013.00467 24391642 PMC3870331

[9] Keats JJ Reiman T Belch AR Pilarski LM. Ten years and counting: so what do we know about t(4;14) (p16;q32) multiple myeloma. Leuk Lymphoma. 2006;47:2289–300. 10.1080/10428190600822128 17107900

[10] Keats JJ Maxwell CA Taylor BJ Hendzel MJ Chesi M Bergsagel PL Overexpression of transcripts originating from the MMSET locus characterizes all t(4;14)(p16;q32)-positive multiple myeloma patients. Blood. 2005;105:4060–9. 10.1182/blood-2004-09-3704 15677557 PMC1895072

[11] Keats JJ Reiman T Maxwell CA Taylor BJ Larratt LM Mant MJ In multiple myeloma, t(4;14)(p16;q32) is an adverse prognostic factor irrespective of FGFR3 expression. Blood. 2003;101:1520–9. 10.1182/blood-2002-06-1675 12393535

[12] Santra M Zhan F Tian E Barlogie B Shaughnessy J Jr. A subset of multiple myeloma harboring the t(4;14)(p16;q32) translocation lacks FGFR3 expression but maintains an IGH/MMSET fusion transcript. Blood. 2003;101:2374–6. 10.1182/blood-2002-09-2801 12433679

[13] Hoyer JD Hanson CA Fonseca R Greipp PR Dewald GW Kurtin PJ. The (11;14)(q13;q32) translocation in multiple myeloma. A morphologic and immunohistochemical study. Am J Clin Pathol. 2000;113:831–7. 10.1309/4W8E-8F4K-BHUP-UBE7 10874884

[14] Gonsalves WI Rajkumar SV Gupta V Morice WG Timm MM Singh PP Quantification of clonal circulating plasma cells in newly diagnosed multiple myeloma: implications for redefining high-risk myeloma. Leukemia. 2014;28:2060–5. 10.1038/leu.2014.98 24618735 PMC4162866

[15] Boersma-Vreugdenhil GR Kuipers J Van Stralen E Peeters T Michaux L Hagemeijer A The recurrent translocation t(14;20)(q32;q12) in multiple myeloma results in aberrant expression of MAFB: a molecular and genetic analysis of the chromosomal breakpoint. Br J Haematol. 2004;126:355–63. 10.1111/j.1365-2141.2004.05050.x 15257707

[16] Ross FM Chiecchio L Dagrada G Protheroe RK Stockley DM Harrison CJ UK Myeloma Forum. The t(14;20) is a poor prognostic factor in myeloma but is associated with long-term stable disease in monoclonal gammopathies of undetermined significance. Haematologica. 2010;95:1221–5. 10.3324/haematol.2009.016329 20410185 PMC2895050

[17] Avet-Loiseau H Gerson F Magrangeas F Minvielle S Harousseau JL Bataille R Intergroupe Francophone du Myélome. Rearrangements of the c-myc oncogene are present in 15% of primary human multiple myeloma tumors. Blood. 2001;98:3082–6. 10.1182/blood.V98.10.3082 11698294

[18] Schuhmacher M Staege MS Pajic A Polack A Weidle UH Bornkamm GW Control of cell growth by c-Myc in the absence of cell division. Curr Biol. 1999;9:1255–8. 10.1016/S0960-9822(99)80507-7 10556095

[19] Prochownik EV. c-Myc: linking transformation and genomic instability. Curr Mol Med. 2008;8:446–58. 10.2174/156652408785747988 18781952

[20] van Riggelen J Yetil A Felsher DW. MYC as a regulator of ribosome biogenesis and protein synthesis. Nat Rev Cancer. 2010;10:301–9. 10.1038/nrc2819 20332779

[21] Beroukhim R Mermel CH Porter D Wei G Raychaudhuri S Donovan J The landscape of somatic copy-number alteration across human cancers. Nature. 2010;463:899–905. 10.1038/nature08822 20164920 PMC2826709

[22] Drach J Schuster J Nowotny H Angerler J Rosenthal F Fiegl M Multiple myeloma: high incidence of chromosomal aneuploidy as detected by interphase fluorescence *in situ* hybridization. Cancer Res. 1995;55:3854–9. 7641204

[23] Manier S Salem K Glavey SV Roccaro AM Ghobrial IM. Genomic aberrations in multiple myeloma. Cancer Treat Res. 2016;169:23–34. 10.1007/978-3-319-40320-5_3 27696256

[24] Yellapantula V Hultcrantz M Rustad EH Wasserman E Londono D Cimera R Correction: Comprehensive detection of recurring genomic abnormalities: a targeted sequencing approach for multiple myeloma. Blood Cancer J. 2020;10:11. Erratum for: Blood Cancer J. 2019;9:101. 10.1038/s41408-020-0279-4 32001687 PMC6992686

[25] Abdi J Rastgoo N Li L Chen W Chang H. Role of tumor suppressor p53 and micro-RNA interplay in multiple myeloma pathogenesis. J Hematol Oncol. 2017;10:169. 10.1186/s13045-017-0538-4 29073933 PMC5659022

[26] Walker BA Mavrommatis K Wardell CP Ashby TC Bauer M Davies F A high-risk, Double-Hit, group of newly diagnosed myeloma identified by genomic analysis. Leukemia. 2019;33:159–70. 10.1038/s41375-018-0196-8 29967379 PMC6326953

[27] Chavan SS He J Tytarenko R Deshpande S Patel P Bailey M Bi-allelic inactivation is more prevalent at relapse in multiple myeloma, identifying RB1 as an independent prognostic marker. Blood Cancer J. 2017;7:e535. 10.1038/bcj.2017.12 28234347 PMC5386330

[28] Walker BA Leone PE Chiecchio L Dickens NJ Jenner MW Boyd KD A compendium of myeloma-associated chromosomal copy number abnormalities and their prognostic value. Blood. 2010;116:e56–65. 10.1182/blood-2010-04-279596 20616218

[29] Calin GA Dumitru CD Shimizu M Bichi R Zupo S Noch E Frequent deletions and down-regulation of micro- RNA genes miR15 and miR16 at 13q14 in chronic lymphocytic leukemia. Proc Natl Acad Sci U S A. 2002;99:15524–9. 10.1073/pnas.242606799 12434020 PMC137750

[30] Hanamura I Stewart JP Huang Y Zhan F Santra M Sawyer JR Frequent gain of chromosome band 1q21 in plasma-cell dyscrasias detected by fluorescence *in situ* hybridization: incidence increases from MGUS to relapsed myeloma and is related to prognosis and disease progression following tandem stem-cell transplantation. Blood. 2006;108:1724–32. 10.1182/blood-2006-03-009910 16705089 PMC1895503

[31] Wu KL Beverloo B Lokhorst HM Segeren CM van der Holt B Steijaert MM Dutch-Belgian Haemato-Oncology Cooperative Study Group (HOVON) Dutch Working Party on Cancer Genetics and Cytogenetics (NWCGC). Abnormalities of chromosome 1p/q are highly associated with chromosome 13/13q deletions and are an adverse prognostic factor for the outcome of high-dose chemotherapy in patients with multiple myeloma. Br J Haematol. 2007;136:615–23. 10.1111/j.1365-2141.2006.06481.x 17223915

[32] Burroughs Garcìa J Eufemiese RA Storti P Sammarelli G Craviotto L Todaro G Role of 1q21 in multiple myeloma: from pathogenesis to possible therapeutic targets. Cells. 2021;10:1360. 10.3390/cells10061360 34205916 PMC8227721

[33] Kawano Y Moschetta M Manier S Glavey S Görgün GT Roccaro AM Targeting the bone marrow microenvironment in multiple myeloma. Immunol Rev. 2015;263:160–72. 10.1111/imr.12233 25510276

[34] Hu J Hu WX. Targeting signaling pathways in multiple myeloma: pathogenesis and implication for treatments. Cancer Lett. 2018;414:214–21. 10.1016/j.canlet.2017.11.020 29174802

[35] Neri A Baldini L Trecca D Cro L Polli E Maiolo AT. p53 gene mutations in multiple myeloma are associated with advanced forms of malignancy. Blood. 1993;81:128–35. 10.1182/blood.V81.1.128.128 8417784

[36] Degirmenci U Wang M Hu J. Targeting aberrant RAS/RAF/MEK/ERK signaling for cancer therapy. Cells. 2020;9:198. 10.3390/cells9010198 31941155 PMC7017232

[37] Walker BA Mavrommatis K Wardell CP Ashby TC Bauer M Davies FE Identification of novel mutational drivers reveals oncogene dependencies in multiple myeloma. Blood. 2018;132:587–97. Erratum in: Blood. 2018;132:1461. 10.1182/blood-2018-08-870022 29884741 PMC6097138

[38] Chapman MA Lawrence MS Keats JJ Cibulskis K Sougnez C Schinzel AC Initial genome sequencing and analysis of multiple myeloma. Nature. 2011;471:467–72. 10.1038/nature09837 21430775 PMC3560292

[39] Xu J Pfarr N Endris V Mai EK Md Hanafiah NH Lehners N Molecular signaling in multiple myeloma: association of RAS/RAF mutations and MEK/ERK pathway activation. Oncogenesis. 2017;6:e337. 10.1038/oncsis.2017.36 28504689 PMC5523069

[40] Letai AG. Diagnosing and exploiting cancer’s addiction to blocks in apoptosis. Nat Rev Cancer. 2008;8:121–32. 10.1038/nrc2297 18202696

[41] Conroy S. Unlicensed and off-label drug use: issues and recommendations. Paediatr Drugs. 2002;4:353–9. 10.2165/00128072-200204060-00002 12038871

[42] Emmerich J Dumarcet N Lorence A. France’s new framework for regulating off-label drug use. N Engl J Med. 2012;367:1279–81. 10.1056/NEJMp1208347 23034018

[43] Stafford RS. Regulating off-label drug use--rethinking the role of the FDA. N Engl J Med. 2008;358:1427–9. 10.1056/NEJMp0802107 18385495

[44] Committee on Drugs. American Academy of Pediatrics. Uses of drugs not described in the package insert (off-label uses). Pediatrics. 2002;110:181–3. 10.1542/peds.110.1.181 12093968

[45] Rayburn WF Farmer KC. Off-label prescribing during pregnancy. Obstet Gynecol Clin North Am. 1997;24:471–8. 10.1016/S0889-8545(05)70317-X 9266573

[46] Gazarian M Kelly M McPhee JR Graudins LV Ward RL Campbell TJ. Off-label use of medicines: consensus recommendations for evaluating appropriateness. Med J Aust. 2006;185:544–8. 10.5694/j.1326-5377.2006.tb00689.x 17115966

[47] Casali PG Executive Committee of ESMO. The off-label use of drugs in oncology: a position paper by the European Society for Medical Oncology (ESMO). Ann Oncol. 2007;18:1923–5. 10.1093/annonc/mdm517 18083693

[48] Boos J. Off label use--label off use? Ann Oncol. 2003;14:1–5. 10.1093/annonc/mdg035 12488285

[49] Fugh-Berman A Melnick D. Off-label promotion, on-target sales. PLoS Med. 2008;5:e210. 10.1371/journal.pmed.0050210 18959472 PMC2573913

[50] Jameson JL Longo DL. Precision medicine--personalized, problematic, and promising. N Engl J Med. 2015;372:2229–34. 10.1056/NEJMsb1503104 26014593

[51] Mulligan G Lichter DI Di Bacco A Blakemore SJ Berger A Koenig E Mutation of NRAS but not KRAS significantly reduces myeloma sensitivity to single-agent bortezomib therapy. Blood. 2014;123:632–9. 10.1182/blood-2013-05-504340 24335104 PMC4123425

[52] Smith D Armenteros E Percy L Kumar M Lach A Herledan G RAS mutation status and bortezomib therapy for relapsed multiple myeloma. Br J Haematol. 2015;169:905–8. 10.1111/bjh.13258 25580780

[53] Walker BA Boyle EM Wardell CP Murison A Begum DB Dahir NM Mutational spectrum, copy number changes, and outcome: results of a sequencing study of patients with newly diagnosed myeloma. J Clin Oncol. 2015;33:3911–20. 10.1200/JCO.2014.59.1503 26282654 PMC6485456

[54] Downward J. Targeting RAS signalling pathways in cancer therapy. Nat Rev Cancer. 2003;3:11–22. 10.1038/nrc969 12509763

[55] Alsina M Fonseca R Wilson EF Belle AN Gerbino E Price-Troska T Farnesyltransferase inhibitor tipifarnib is well tolerated, induces stabilization of disease, and inhibits farnesylation and oncogenic/tumor survival pathways in patients with advanced multiple myeloma. Blood. 2004;103:3271–7. 10.1182/blood-2003-08-2764 14726402

[56] Lito P Pratilas CA Joseph EW Tadi M Halilovic E Zubrowski M Relief of profound feedback inhibition of mitogenic signaling by RAF inhibitors attenuates their activity in BRAFV600E melanomas. Cancer Cell. 2012;22:668–82. 10.1016/j.ccr.2012.10.009 23153539 PMC3713778

[57] Heidorn SJ Milagre C Whittaker S Nourry A Niculescu-Duvas I Dhomen N Kinase-dead BRAF and oncogenic RAS cooperate to drive tumor progression through CRAF. Cell. 2010;140:209–21. 10.1016/j.cell.2009.12.040 20141835 PMC2872605

[58] Garnett MJ Rana S Paterson H Barford D Marais R. Wild-type and mutant B-RAF activate C-RAF through distinct mechanisms involving heterodimerization. Mol Cell. 2005;20:963–9. 10.1016/j.molcel.2005.10.022 16364920

[59] Lunghi P Giuliani N Mazzera L Lombardi G Ricca M Corradi A Targeting MEK/MAPK signal transduction module potentiates ATO-induced apoptosis in multiple myeloma cells through multiple signaling pathways. Blood. 2008;112:2450–62. 10.1182/blood-2007-10-114348 18583568

[60] Heuck CJ Jethava Y Khan R van Rhee F Zangari M Chavan S Inhibiting MEK in MAPK pathway-activated myeloma. Leukemia. 2016;30:976–80. 10.1038/leu.2015.208 26228812 PMC4832073

[61] Andrulis M Lehners N Capper D Penzel R Heining C Huellein J Targeting the BRAF V600E mutation in multiple myeloma. Cancer Discov. 2013;3:862–9. 10.1158/2159-8290.CD-13-0014 23612012

[62] Sharman JP Chmielecki J Morosini D Palmer GA Ross JS Stephens PJ Vemurafenib response in 2 patients with posttransplant refractory BRAF V600E-mutated multiple myeloma. Clin Lymphoma Myeloma Leuk. 2014;14:E161–3. 10.1016/j.clml.2014.06.004 24997557

[63] Salama AKS Li S Macrae ER Park JI Mitchell EP Zwiebel JA Dabrafenib and trametinib in patients with tumors with *BRAFV600E* mutations: results of the NCI-MATCH trial subprotocol H. J Clin Oncol. 2020;38:3895–904. 10.1200/JCO.20.00762 32758030 PMC7676884

[64] Guo C Chénard-Poirier M Roda D de Miguel M Harris SJ Candilejo IM Intermittent schedules of the oral RAF-MEK inhibitor CH5126766/VS-6766 in patients with RAS/RAF-mutant solid tumours and multiple myeloma: a single-centre, open-label, phase 1 dose-escalation and basket dose-expansion study. Lancet Oncol. 2020;21:1478–88. Erratum in: Lancet Oncol. 2021;22:e42. 10.1016/S1470-2045(20)30464-2 33128873

[65] Emery CM Monaco KA Wang P Balak M Freeman A Meltzer J BRAF-inhibitor associated MEK mutations increase RAF-dependent and -independent enzymatic activity. Mol Cancer Res. 2017;15:1431–44. 10.1158/1541-7786.MCR-17-0211 28655712

[66] Wagle N Emery C Berger MF Davis MJ Sawyer A Pochanard P Dissecting therapeutic resistance to RAF inhibition in melanoma by tumor genomic profiling. J Clin Oncol. 2011;29:3085–96. 10.1200/JCO.2010.33.2312 21383288 PMC3157968

[67] Turke AB Song Y Costa C Cook R Arteaga CL Asara JM MEK inhibition leads to PI3K/AKT activation by relieving a negative feedback on ERBB receptors. Cancer Res. 2012;72:3228–37. 10.1158/0008-5472.CAN-11-3747 22552284 PMC3515079

[68] Long GV Stroyakovskiy D Gogas H Levchenko E de Braud F Larkin J Combined BRAF and MEK inhibition *versus* BRAF inhibition alone in melanoma. N Engl J Med. 2014;371:1877–88. 10.1056/NEJMoa1406037 25265492

[69] Raab MS Giesen N Scheid C Besemer B Miah K Benner A Safety and preliminary efficacy results from a phase II study evaluating combined BRAF and MEK inhibition in relapsed/refractory multiple myeloma (rrMM) patients with activating BRAF V600E mutations: the GMMG-Birma trial. Blood. 2020;136:44–5. 10.1182/blood-2020-142600

[70] Haertle L Barrio S Simicek M Munawar U Sanchez R Bittrich M Mechanisms of proteasome inhibitor resistance selected by clonal evolution in multiple myeloma. Blood. 2019;134:4349. 10.1182/blood-2019-130847

[71] Cory S Adams JM. The Bcl2 family: regulators of the cellular life-or-death switch. Nat Rev Cancer. 2002;2:647–56. 10.1038/nrc883 12209154

[72] Chen L Willis SN Wei A Smith BJ Fletcher JI Hinds MG Differential targeting of prosurvival Bcl-2 proteins by their BH3-only ligands allows complementary apoptotic function. Mol Cell. 2005;17:393–403. 10.1016/j.molcel.2004.12.030 15694340

[73] Letai A Bassik MC Walensky LD Sorcinelli MD Weiler S Korsmeyer SJ. Distinct BH3 domains either sensitize or activate mitochondrial apoptosis, serving as prototype cancer therapeutics. Cancer Cell. 2002;2:183–92. 10.1016/S1535-6108(02)00127-7 12242151

[74] Cheng EH Wei MC Weiler S Flavell RA Mak TW Lindsten T BCL-2, BCL-X(L) sequester BH3 domain-only molecules preventing BAX- and BAK-mediated mitochondrial apoptosis. Mol Cell. 2001;8:705–11. 10.1016/S1097-2765(01)00320-3 11583631

[75] Ola MS Nawaz M Ahsan H. Role of Bcl-2 family proteins and caspases in the regulation of apoptosis. Mol Cell Biochem. 2011;351:41–58. 10.1007/s11010-010-0709-x 21210296

[76] Li P Nijhawan D Budihardjo I Srinivasula SM Ahmad M Alnemri ES Cytochrome c and dATP- dependent formation of Apaf-1/caspase-9 complex initiates an apoptotic protease cascade. Cell. 1997;91:479–89. 10.1016/S0092-8674(00)80434-1 9390557

[77] Gentile M Petrungaro A Uccello G Vigna E Recchia AG Caruso N Venetoclax for the treatment of chronic lymphocytic leukemia. Expert Opin Investig Drugs. 2017;26:1307–16. 10.1080/13543784.2017.1386173 28972395

[78] Costa LJ Davies FE Monohan GP Kovacsovics T Burwick N Jakubowiak A Phase 2 study of venetoclax plus carfilzomib and dexamethasone in patients with relapsed/refractory multiple myeloma. Blood Adv. 2021;5:3748–59. 10.1182/bloodadvances.2020004146 34470049 PMC8679663

[79] Villalobos-Ortiz M Ryan J Mashaka TN Opferman JT Letai A. BH3 profiling discriminates on-target small molecule BH3 mimetics from putative mimetics. Cell Death Differ. 2020;27:999–1007. 10.1038/s41418-019-0391-9 31332296 PMC7205860

[80] Bhola PD Ahmed E Guerriero JL Sicinska E Su E Lavrova E High-throughput dynamic BH3 profiling may quickly and accurately predict effective therapies in solid tumors. Sci Signal. 2020;13:eaay1451. 10.1126/scisignal.aay1451 32546544 PMC8023011

[81] Carné Trécesson S Souazé F Basseville A Bernard AC Pécot J Lopez J BCL-XL directly modulates RAS signalling to favour cancer cell stemness. Nat Commun. 2017;8:1123. 10.1038/s41467-017-01079-1 29066722 PMC5654832

[82] Konopleva M Contractor R Tsao T Samudio I Ruvolo PP Kitada S Mechanisms of apoptosis sensitivity and resistance to the BH3 mimetic ABT-737 in acute myeloid leukemia. Cancer Cell. 2006;10:375–88. 10.1016/j.ccr.2006.10.006 17097560

[83] Touzeau C Dousset C Le Gouill S Sampath D Leverson JD Souers AJ The Bcl-2 specific BH3 mimetic ABT-199: a promising targeted therapy for t(11;14) multiple myeloma. Leukemia. 2014;28:210–2. 10.1038/leu.2013.216 23860449 PMC3887407

[84] Kumar S Kaufman JL Gasparetto C Mikhael J Vij R Pegourie B Efficacy of venetoclax as targeted therapy for relapsed/refractory t(11;14) multiple myeloma. Blood. 2017;130:2401–9. 10.1182/blood-2017-06-788786 29018077

[85] Gupta VA Barwick BG Matulis SM Shirasaki R Jaye DL Keats JJ Venetoclax sensitivity in multiple myeloma is associated with B-cell gene expression. Blood. 2021;137:3604–15. 10.1182/blood.2020007899 33649772 PMC8462405

[86] Todoerti K Taiana E Puccio N Favasuli V Lionetti M Silvestris I Transcriptomic analysis in multiple myeloma and primary plasma cell leukemia with t(11;14) reveals different expression patterns with biological implications in venetoclax sensitivity. Cancers (Basel). 2021;13:4898. 10.3390/cancers13194898 34638381 PMC8508148

[87] Murphy TL Tussiwand R Murphy KM. Specificity through cooperation: BATF–IRF interactions control immune-regulatory networks. Nat Rev Immunol. 2013;13:499–509. 10.1038/nri3470 23787991

[88] Moreau P Chanan-Khan A Roberts AW Agarwal AB Facon T Kumar S Promising efficacy and acceptable safety of venetoclax plus bortezomib and dexamethasone in relapsed/refractory MM. Blood. 2017;130:2392–400. 10.1182/blood-2017-06-788323 28847998

[89] Kaufman JL Gasparetto C Schjesvold FH Moreau P Touzeau C Facon T Targeting BCL-2 with venetoclax and dexamethasone in patients with relapsed/refractory t(11;14) multiple myeloma. Am J Hematol. 2021;96:418–27. 10.1002/ajh.26083 33368455 PMC7986778

[90] Matulis SM Gupta VA Nooka AK Hollen HV Kaufman JL Lonial S Dexamethasone treatment promotes Bcl-2 dependence in multiple myeloma resulting in sensitivity to venetoclax. Leukemia. 2016;30:1086–93. 10.1038/leu.2015.350 26707935 PMC4874660

[91] Kumar SK Harrison SJ Cavo M de la Rubia J Popat R Gasparetto C Venetoclax or placebo in combination with bortezomib and dexamethasone in patients with relapsed or refractory multiple myeloma (BELLINI): a randomised, double-blind, multicentre, phase 3 trial. Lancet Oncol. 2020;21:1630–42. 10.1016/S1470-2045(20)30525-8 33129376

[92] Mateos MV Moreau P Dimopoulos MA Hong WJ Cooper S Yu Y A phase III, randomized, multicenter, open-label study of venetoclax or pomalidomide in combination with dexamethasone in patients with t(11;14)-positive relapsed/refractory multiple myeloma [abstract]. J Clin Oncol. 2020;38:TPS8554. 10.1200/JCO.2020.38.15_suppl.TPS8554

[93] Tausch E Close W Dolnik A Bloehdorn J Chyla B Bullinger L Venetoclax resistance and acquired *BCL2* mutations in chronic lymphocytic leukemia. Haematologica. 2019;104:e434–7. 10.3324/haematol.2019.222588 31004028 PMC6717583

[94] Matulis SM Gupta VA Neri P Bahlis NJ Maciag P Leverson JD Functional profiling of venetoclax sensitivity can predict clinical response in multiple myeloma. Leukemia. 2019;33:1291–6. 10.1038/s41375-018-0374-8 30679802 PMC6891824

